# Unsupervised learning-enabled pulsed infrared thermographic microscopy of subsurface defects in stainless steel

**DOI:** 10.1038/s41598-024-64214-1

**Published:** 2024-06-27

**Authors:** Xin Zhang, Tianyang Fang, Jafar Saniie, Sasan Bakhtiari, Alexander Heifetz

**Affiliations:** 1 Nuclear Science and Engineering Division, Argonne National Laboratory, Lemont, IL 60439 USA; 2 Department of Electrical and Computer Engineering, Illinois Institute of Technology, Chicago, IL 60616 USA

**Keywords:** Imaging and sensing, Computer science, Imaging techniques

## Abstract

Metallic structures produced with laser powder bed fusion (LPBF) additive manufacturing method (AM) frequently contain microscopic porosity defects, with typical approximate size distribution from one to 100 microns. Presence of such defects could lead to premature failure of the structure. In principle, structural integrity assessment of LPBF metals can be accomplished with nondestructive evaluation (NDE). Pulsed infrared thermography (PIT) is a non-contact, one-sided NDE method that allows for imaging of internal defects in arbitrary size and shape metallic structures using heat transfer. PIT imaging is performed using compact instrumentation consisting of a flash lamp for deposition of a heat pulse, and a fast frame infrared (IR) camera for measuring surface temperature transients. However, limitations of imaging resolution with PIT include blurring due to heat diffusion, sensitivity limit of the IR camera. We demonstrate enhancement of PIT imaging capability with unsupervised learning (UL), which enables PIT microscopy of subsurface defects in high strength corrosion resistant stainless steel 316 alloy. PIT images were processed with UL spatial–temporal separation-based clustering segmentation (STSCS) algorithm, refined by morphology image processing methods to enhance visibility of defects. The STSCS algorithm starts with wavelet decomposition to spatially de-noise thermograms, followed by UL principal component analysis (PCA), fine-tuning optimization, and neural learning-based independent component analysis (ICA) algorithms to temporally compress de-noised thermograms. The compressed thermograms were further processed with UL-based graph thresholding K-means clustering algorithm for defects segmentation. The STSCS algorithm also includes online learning feature for efficient re-training of the model with new data. For this study, metallic specimens with calibrated microscopic flat bottom hole defects, with diameters in the range from 203 to 76 µm, were produced using electro discharge machining (EDM) drilling. While the raw thermograms do not show any material defects, using STSCS algorithm to process PIT images reveals defects as small as 101 µm in diameter. To the best of our knowledge, this is the smallest reported size of a sub-surface defect in a metal imaged with PIT, which demonstrates the PIT capability of detecting defects in the size range relevant to quality control requirements of LPBF-printed high-strength metals.

## Introduction

### Overview of nondestructive evaluation for metal additive manufacturing

Additive Manufacturing (AM) is an emerging method for the fabrication of complex shape custom structures^1^. AM of high-strength corrosion-resistant metallic alloys, such as stainless steel 316 (SS316) and Inconel 718 (IN718) with melting temperatures in the range of 1370 to 1430 °C, is currently based on the Laser Powder Bed Fusion (LPBF) method^2^. LPBF involves complex processes governed by recoil pressure and Marangoni convection, which include rapid melting of microscopic metallic powder particles and cooling of the melt pool without well-defined boundary conditions^3^. Oscillations in the surface of the melt pool caused by rapid heating and cooling cause powder ejection and splattering. As a result, porosity defects can be introduced into AM parts due to incomplete melting of powder particles or insufficient overlap of melt pools^4^. Typical porosity defects observed in LPBF manufacturing consist of microscopic spheroidal-shape keyhole pores caused by excessive laser power, irregular-shape lack of fusion (LOF) pores caused by insufficient laser power, and spherical gas pores caused by gas trapped in the solidifying melt pool^5–8^. Typical size distribution for defects in LPBF-printed metals is approximately between one and 100 microns. For AM metallic structures exposed to harsh environment, such as high temperature and ionizing radiation in a nuclear reactor, material defects could result in lower-than-expected failure resistance or fatigue life^9–11^. The likelihood of fatigue crack initiation in AM metals depends on factors such as the size and shapes of pores, proximity to the surface, and orientation relative to the surface plane^7,8^. In addition, corrosion damage to AM metallic structures could be accelerated by corrosion front encountering sub-surface pores in the metal^12,13^. Therefore, nondestructive evaluation (NDE) is needed to verify the integrity of metallic AM structures before they enter service in harsh environment^14^. Furthermore, because long-term behavior of AM metals in a nuclear environment is not known, condition of AM structures needs to be monitored through in-service NDE inspections.

Currently, there is no accepted method for qualification of AM metallic components^15^. During manufacturing, material flaws can, in principle, be detected using X-ray radiography^16^ or thermography ^17^. X-ray imaging has been used primarily in proof-of-principle studies for basic understanding of physical processes in LPBF. In-situ thermography with an infrared (IR) camera installed in a metal 3D printer aims to detect relative variation of the melt pool cooling rates, which could be attributed to formation of pores. However, because of high variance in the background temperature during unsteady heat conduction in a random media powder bed system without well-defined boundaries, real-time flaw detection with thermography is not trivial. Moreover, microscopic pores appearing in a given printed layer could fuse in re-melting during printing of the subsequent layer.

There exist several NDE approaches to image pores in a printed metallic structure. In principle, X-ray Computed Tomography (XCT) allows for non-contact high-resolution imaging of metals^18,19^. However, XCT requires structures with symmetric bodies of revolution shapes (spheres and cylinders). Because of attenuation of X-rays in SS316 and IN718, penetration depth in practice is typically limited to distances on the order of a few centimeters. In general, XCT imaging resolution decreases with increasing structure size. Since AM metallic structures usually involve arbitrary size structures with complex shapes without rotational symmetry, XCT faces challenges in imaging internal defects in actual AM structures. Neutron tomography allows for longer penetration depth, and can be used for imaging of pores in AM structures^20^. However, a source of neutron beam is typically a nuclear reactor, which limits accessibility of this approach. In addition, neutron activation of SS316 is a potential concern. Ultrasonic testing (UT) is a widely used method for detection of microscopic defects in metals, which is scalable to arbitrary structure sizes and surface shapes^21,22^. However, UT requires direct contact of a probe with material surface, which is difficult to achieve with AM structures which have rough surfaces due to melt pool oscillations. Eddy Current (EC) imaging is a common method in nuclear structures NDE because EC uses non-contact inductive probes resilient to harsh environments. However, EC measurements can be affected by material surface shape and temperature irregularities^23^. In addition, high resolution imaging in both UT and EC typically requires time-consuming raster scanning with a single probe.

### Overview of active infrared thermography imaging for nondestructive evaluation in metal additive manufacturing

Pulsed Infrared Thermography (PIT) offers several advantages for nondestructive detection of subsurface pores in AM structures because this method is non-contact, one-sided, and scalable to arbitrary structure size. Pulsed or flash thermography is an active thermography method, in which a thermal pulse is deposited on a material surface with a flash lamp. As heat diffuses into the material bulk, surface temperature transients are recorded with a fast frame infrared (IR) camera via detection of blackbody radiation^24–27^. An air-filled pore has much smaller thermal conductivity than that of the surrounding metallic matrix. Thermal resistance of the pores results in a relatively slower local surface temperature decay above the internal material defect, so that presence of internal pores in a metal is revealed through transient appearance of temperature “hot spots” on the material surface. Thus, information about internal defects can be obtained from the data cube of time-resolved sequential frames of thermal images of material surface temperature. While there exist other approaches to active thermography, such as lock-in thermography or flying spot thermography, PIT has the advantage of relative simplicity of experimental measurements^28–31^.

Detection of thermal signatures is typically performed with mid-wave infrared (MWIR) camera with 3–5 µm spectral range, with high-end cooled semiconductor cameras having sensitivities limited by noise equivalent temperature difference (NETD) of 20 mK. In principle, diffraction limited detection of 5 µm features is possible. However, because heat diffusion is not confined to 1D, thermal image of the flaw suffers from edge blurring and reduction of thermal intensity contrast. In addition, thermal image quality is affected by non-uniform deposition of thermal pulse energy due to material surface roughness, and non-uniform surface emissivity. Imaging microscopic defects in metals requires analyzing weak thermal signatures comparable in amplitude to IR camera NETD. Recently investigated approaches for characterization of material defects in PIT data include analytical and computational models^32–34^, virtual waves^35,36^, machine learning (ML) methods^37–39^, and depth reconstruction of thermal effusivity^40–43^. However, these studies demonstrate applications of thermography data processing algorithms to experimental millimeter-size defects, which are an order of magnitude larger than the length scales of pores in AM metals.

To improve visibility of defects in PIT images, we have recently developed several unsupervised learning (UL) methods, including several neural learning blind source separation (NLBSS) algorithms which learn latent principal patterns in data with minimal human supervision^44–49^. In prior studies performed with imaging metallic specimens with calibrated defects in millimeter to sub-millimeter size, we have obtained the most promising results using the spatial temporal blind source separation (STBSS) algorithm^45^, which consists of wavelet denoising followed by the principal component analysis (PCA)^50^ and independent component analysis (ICA)^51^. Enhanced visibility of material defects in thermal images is obtained by training STBSS to learn the principal features in thermography data cube, and to remove unimportant information^52,53^. In this paper, we demonstrate an experimental PIT detection of microscopic subsurface defects in stainless steel using spatial–temporal separation-based clustering segmentation (STSCS) algorithm. Compared to previously reported STBSS and NLBSS algorithms, we have introduced fine tuning with optimization, UL-based graph thresholding to perform defect segmentation in thermal images, and on-line learning features into the STSCS algorithm. The fine-tuning process with optimizations consists of using adaptive learning rate method and the hyperbolic tangent as nonquadratic function for better learning convergence to improve ICA training performance. This enables STSCS detection of microscopic defects as small as 101 µm using weak PIT thermal signatures that are close to noise level. To enhance visibility of the defects with weak thermal features that are close to the noise level, the defects in compressed thermograms were segmented with a UL-based graph thresholding K-means clustering algorithm refined with morphology image processing methods to define edges. We also added an online learning consisting of incremental learning mechanism that performs stochastic training by dividing the PIT data into small batches^49^. Online learning allows the STSCS model to efficiently acquire new information whenever a new training set becomes available, while preserving the knowledge learned from the previous training data sets^49^.

To produce a set of calibrated material defects resembling pores in AM structures, we developed metallic plates with flat bottom hole (FBH) or blind hole defects. Such specimens are flat on one side and have a set of cylindrical holes that stop short at various depths below the flat surface. Unlike the pores in AM structures, the FBH defects are not completely enclosed by the metallic matrix. However, from the physics of heat diffusion perspective, PIT detection of the two defects is equivalent. The FBH represents the top of a pore, and detection of either FBH or internal pore defect occurs when the diffusing heat pulse encounters a local air gap. Specimens in this study consisted of SS316 plates with microscopic FBH defects produced using electrical discharge machining (EDM) drilling. The FBH defects range in diameters from 200 to 75 µm, with depths below the flat plate surface in the range from 500 to 127 µm. An alternative approach of imprinting similar size internal calibrated defects with LPBF is challenging because this would require selective melting of individual microscopic metallic powder particles. In prior studies we have created LPBF plates containing hemispherical defects with diameters larger than 500 µm, which consisted of pockets of un-sintered powder particles. However, thermal conductivity of such defects is higher than those of pores filled with air. In imaging the SS316 plates with PIT, the raw recorded thermograms did not display any features corresponding to defects. However, processing the PIT data cube with STSCS algorithm has revealed defects as small as 101 µm in diameter. To the best of our knowledge, these are the smallest reported defects in a metal with PIT, which demonstrates PIT capability in nondestructive detection of defects in the size range relevant to quality control requirements of LPBF-printed high-strength metals.

## Results

### Visualization of thermal images of defects with computer simulations

To investigate microscopy of subsurface internal defects in a metal, we imaged two SS316 plates containing calibrated microscopic FBH defects. One side of each plate was flat, and the other side contained a matrix pattern of FBH’s. The flat side of each plate was painted with Krylon flat black paint. Painting the specimens increases absorption of the incident optical flash and normalizes PIT measurement for specimens with different surfaces. Plate 1 with 32.9 mm × 32.9 mm × 1.4 mm dimensions contained FBH’s with diameters 203 µm, 178 µm, 152 µm, and 127 µm. The FBH defects were located at depths 508 µm, 381 µm, 254 µm, and 127 µm, as measured from the top of the FBH to the top of the flat side of the plate. Plate 2 with 32.9 mm × 30.4 mm × 0.5 mm dimensions contained FBH’s with diameters 152 µm, 127 µm, 101 µm, and 76 µm. The FBH’s were located at depths 381 µm, 254 µm, and 127 µm.

To illustrate the challenges in PIT imaging of microscopic subsurface defects in stainless steel, we perform computer simulations of surface temperature transients with COMSOL modeling of heat transfer in solids. Note that the COMSOL simulation is not an exact model of the PIT experiment described in the Methods section. Instead, the goal of COMSOL modelling is to provide approximate estimates of the magnitude of expected surface temperature contrast for the “hot spots” in Plates 1 and 2. With the user-defined mesh setting with the maximum element size of 100 µm and minimum element size of 0.1 µm, COMSOL model automatically developed the optimal meshing scheme consisting of triangular grids. Thermal insulating boundary conditions were imposed on the plates. Heat flux of 70 MW/m^2^ was deposited for 5 ms on the flat surface of each plate. The parameters of the heat flux were chosen to for COMSOL simulations to match the experimentally observed maximum temperatures of 673 K (≈400C) of the plates immediately after heat pulse deposition. The plates were assumed to be initially at 293 K (room temperature). Temperature-dependent thermophysical properties of stainless steel 316 in COMSOL material library were used for heat transfer calculations. Temperature on the surfaces of both plates was sampled every 5 ms.

**Figure 1:**
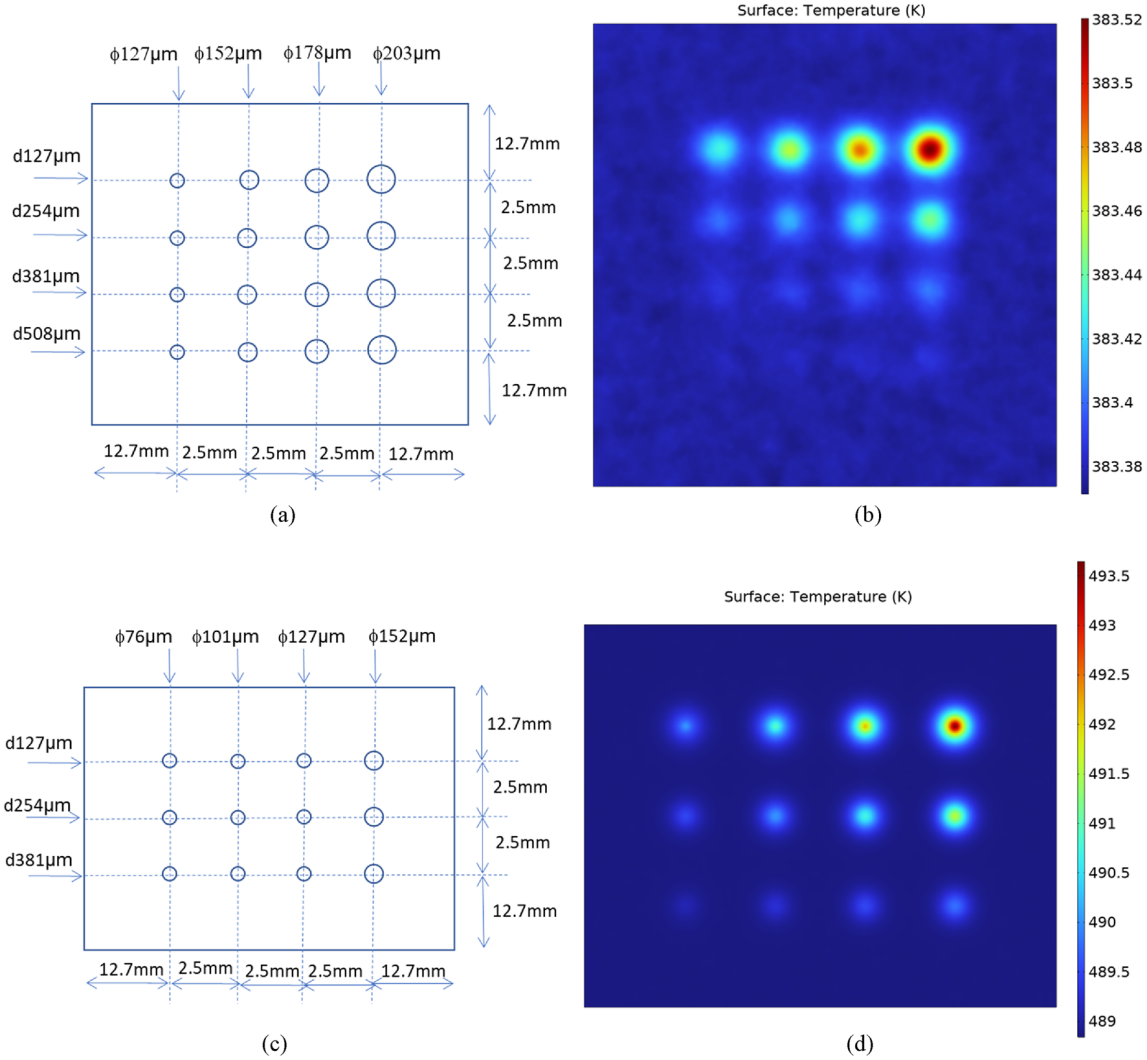
(**a**) Design pattern of FBH defects in Plate 1 (1.4 mm-thick SS316) indicating diameters of defects *ɸ* and depths below surface *d*. (**b**) Example of surface temperature from COMSOL heat transfer simulations of Plate 1 at *t* = 0.03 s after heat pulse deposition. (**c**) Design pattern of FBH defects in Plate 2 (0.5 mm-thick SS316) indicating diameters of defects *ɸ* and depths below surface *d*. (**d**) Example of surface temperature from COMSOL heat transfer simulations of Plate 2 at *t* = 0.01 s after heat pulse deposition.

The design patterns of FBH defects in Plates 1 and 2, indicating diameters of the defects *ɸ* and corresponding depths *d*, are shown in Fig. 1a,c, respectively. Note that the drawings are not-to-scale. The example of surface temperature snapshot from COMSOL simulations visualizing the temperature “hot spots” at *t* = 0.03 s after heat pulse deposition for Plate 1, and at *t* = 0.01 s after heat pulse deposition for Plate 2 are shown in Fig. 1b,d, respectively. Surface temperature frames from the COMSOL computer simulation were chosen so that “hot spots” visualizing all defects in the plate could be visible in the frame. The dynamic ranges of surface temperature for the largest and closest to surface defects for Plates 1 and 2 are 0.14 K and 3.5 K, respectively. In principle, 20 mK NETD of the IR camera is sufficient for imaging with such temperature dynamic ranges. The thermal contrast of defects is decreasing with decreasing defect size and increasing depth. However, it is anticipated that PIT measurements of Plates 1 and 2, contaminated by experimental noise, and with non-ideal insulating boundary conditions, it would be difficult to observe material defects in the raw measurements.

### PIT imaging and reconstruction of defects with STSCS algorithm

Plates 1 and 2 were imaged with PIT system using FLIR X8501SC camera. In two separate experiments, the plates were taped to a piece of cardboard, which is a relatively good thermal insulator. Thermal pulse was delivered to the plates by discharging a 6.4 kJ capacitor with a 2 ms time constant through Balcar white light flash lamp. Plate 1 was imaged with the frame rate of 220 Hz and frame window size of 752 × 704 pixels, using 50-mm lens with 1.5in extender rings to obtain 18 µm/pixel spatial resolution. Total imaging time was 2.73 s, with 600 total frames recorded in the data set. Plate 2 was imaged with the frame rate of 240 Hz and with window size of 728 × 960 pixels, using 50-mm lens with 1.75in extender rings to obtain 11 µm/pixel spatial resolution. Total imaging time was 2.5 s, with 600 total frames recorded in the data set. Each dataset forms a 3D data cube and is converted into the condensed 2D data matrix for training. The 2D data matrix consists of the total number of pixels in each thermogram into a singular vector with the time-dependent information into columns. The sizes of 3D data cube for Plate 1 and Plate 2 are: 752 × 704 × 600 and 728 × 960 × 600 respectively. Imaging settings on the IR camera were determined in several experimental trials, with data recording followed by STSCS algorithm reconstruction.

**Figure 2:**
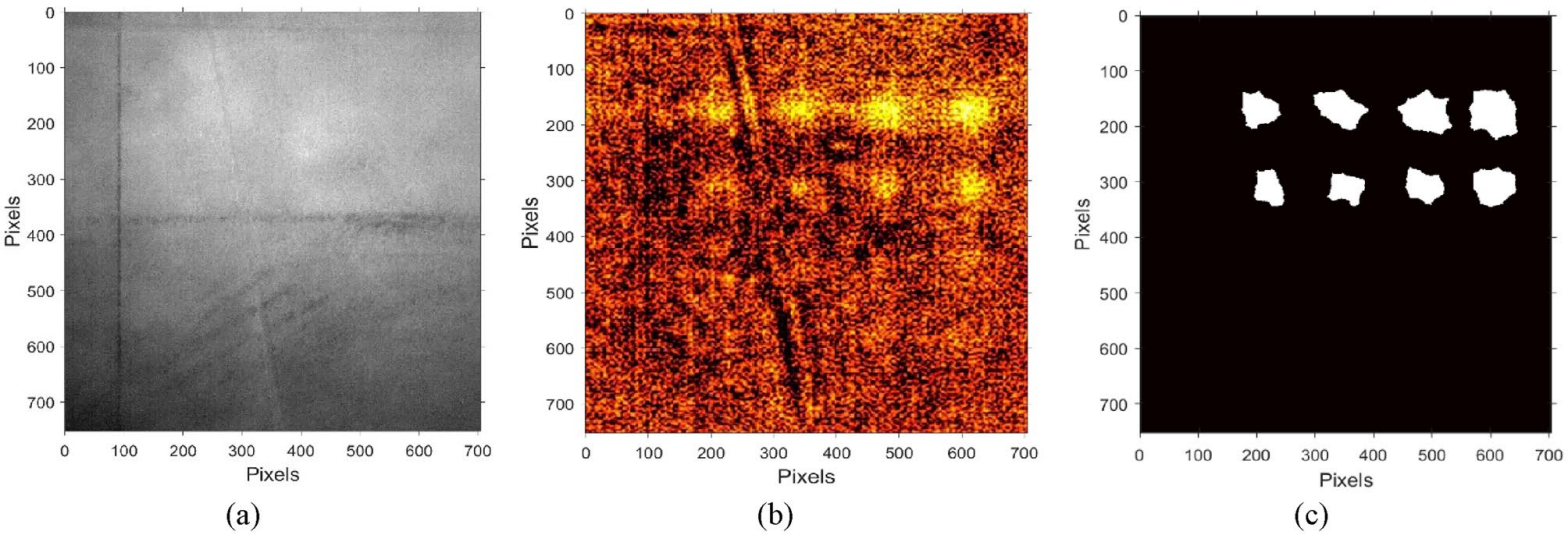
Imaging defects in 1.4 mm-thick SS316 Plate 1 with IR camera window size 752 × 704 pixels and frame rate 220 Hz. Imaging resolution is 18 µm/pixel. Smallest visible FBH is 127 µm-diameter located at 254 µm depth. (**a**) Featureless raw thermography frame *t* = 0.05 s after the flash, with the average temperature of 302 K. (**b**) STSCS thermal image reconstruction. (**c**) STSCS defects segmentation.

**Figure 3:**
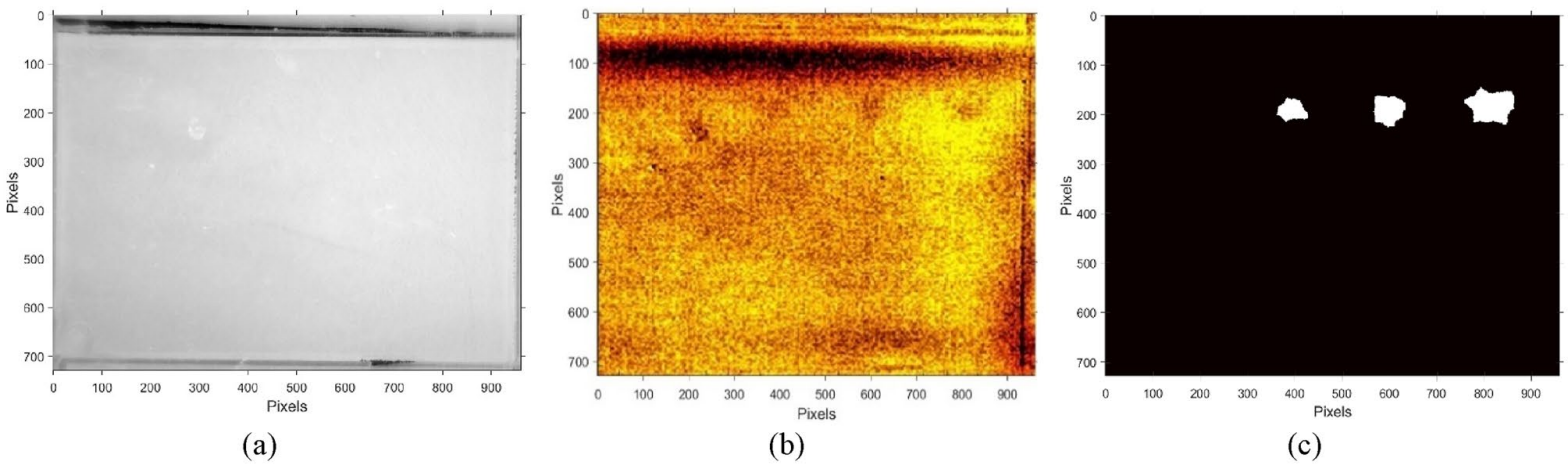
Imaging defects in Plate 2 0.5 mm-thick SS316 Plate 2 with IR camera frame size 728 × 960 pixels and 240 Hz frame rate. Imaging resolution is 11 µm/pixel. Smallest visible defect is 101 µm-diameter FBH located at 127 µm depth. (**a**) Featureless thermography frame *t* = 0.025 s after the flash, with approximately 306 K average temperature. (**b**) STSCS thermal image reconstruction. (**c**) STSCS defects segmentation.

The raw (as-measured) thermography frames did not reveal the presence of material defects in either plate. A featureless thermography frame of Plate 1, recorded at *t* = 0.05 s after flash, with average temperature of 302 K, is shown in Fig. 2a. A featureless thermography frame of Plate 2, recorded at *t* = 0.025 s after flash, with average temperature of 306 K, is shown in Fig. 3a. As compared to COMSOL simulations in Fig. 1, experimental measurements suffer from imaging contrast losses. For the same time after heat pulse deposition, the temperatures of both plates observed during the experiment are significantly lower than those of COMSOL simulations with thermally insulated boundary conditions. Temperature differences between experiment and simulation could be attributed to presence of heat sinks during the experiment. Another source of thermal imaging losses can be attributed to non-uniform heating of the specimen with the flash lamp pulse, since the plane of the specimen is normal to the IR camera field of view, but at an angle to the incident light flux from the flash lamp. In addition, absorption of heat on the specimen surface is facilitated by the paint, which has non-uniform surface density due to uneven drying. Another source of thermal imaging losses could be non-uniform surface emissivity due to scratches and surface roughness of the specimens.

Reconstructions of thermal images with the STSCS algorithm revealing the presence of material defects in Plates 1 and 2 are shown in Figs. 2b and 3b, respectively. The STSCS algorithm starts with wavelet decomposition to spatially de-noise PIT images. This is followed by PCA and neural learning-based ICA algorithms to temporally compress de-noised thermograms. The number of PC’s and independent components was optimized to retain sufficient PIT information and discretize each independent thermography signal for defects detection. The result was that we selected 30 PC’s and 10 independent components as output neurons for reconstruction of material defects in images.

Results of segmentation of defects in thermal images of Plates 1 and 2 with STSCS algorithm are shown in Figs. 2c and 3c, respectively. The compressed thermograms were processed with a UL-based graph thresholding K-means clustering algorithm for defect segmentation. To compensate for negative outcomes from the thresholding process, we refined defect segmentation by morphology image processing methods to improve visibility of defects with weak thermal features that are close to the noise level. The smallest visible defect in Plate 1 in Fig. 1c is the 127 µm-diameter FBH located at 254 µm depth. The smallest visible defect in Plate 2 is the 101 µm-diameter FBH located at 127 µm depth. Defects that were smaller and/or located deeper below the plate surface were not observed. As shown in COMSOL simulations in Fig. 1c,d, thermal signals of these defects are expected to be relatively weaker. Note that the 127 µm defect was detected at 254 µm depth in a 1.4 mm-thick Plate 1, but not detected at this depth in a 0.5 mm-thick Plate 2. The two plates have approximately the same surface areas, but the thickness of Plate 2 is almost three times smaller than for Plate 1. We believe the reason for this is faster decay of thermal transients in the thinner Plate 2, which allows for recording of fewer thermography frames where the thermal signal of the defect is above the noise level.

## Discussion

In this work, we use the defects design pattern, shown in Fig. 1a,c, as the ground truth for evaluating the STSCS algorithm performance. A common metric for the accuracy of defects detection in NDE applications is the F-score ^45^. This metric indicates how well the STSCS algorithm can separate the true signals from noise. The F-score is proportional to the number of defects that are detected out of the total number of calibrated defects in the specimen. The values of the F-score are between 0 and 1, with the higher number indicating better accuracy.

1 $$ F_{score} = \frac{{\left( {\beta + 1} \right) \times \left( {Precision \times Recall} \right)}}{{\beta^{2} \times \left( {Precision + Recall} \right)}} $$

2 $$ Precision = T_{p} /\left( {T_{p} + F_{p} } \right) $$

3 $$ Recall = T_{p} /\left( {T_{p} + F_{n} } \right) $$

In equations above, *Precision* indicates the ratio of true positive to the sum of true positive and false positive detections. *Recall* indicates the ratio of true positive to the sum of true positive and false negative detections. *T*_*p*_ = true positive, *F*_*p*_ = false positive, *F*_*n*_ = false negative. *β* is the tradeoff parameter, which determines whether Precision or Recall is more important. In this study, we set *β* = 2, which indicates that Recall is prioritized over Precision. This decision aims to enhance defects detectability to minimize the risk of structural failure. In practice, PIT indication of a material flaw could be used a cue for additional examination of the structure with other methods.

As seen in Figs. 2 and 3, STSCS algorithm detects 8 out of 16 FBH defects in Plate 1, and 3 out of 12 FBH defects in Plate 2. No defects that do not exist were suggested by the STSCS algorithm. Table 1 summarizes the values of the parameters in the STSCS algorithm performance analysis.

**Table 1:** Summary of STSCS performance scores.

Parameter	Plate 1	Plate 2
*T*_p_	8	3
*F*_p_	0	0
*F*_n_	8	9
*Precision*	1	1
*Recall*	0.5	0.25
*F-score*	0.25	0.15

While the F-scores are relatively low, it should be noted that sizes of the FBH defects in this study are an order of magnitude smaller than typical sizes of calibrated FBH defects reported in existing literature. To the best of our knowledge, these are the smallest reported defects in a metal with PIT, which demonstrates PIT capability in nondestructive detection of defects in the size range relevant to quality control requirements of LPBF-printed high-strength metals. The main sources of error in the STSCS algorithm performance are related to false negative, or inability to detect smaller and deeper defects. This is caused by the underlying heat diffusion physics of image formation. Because of limited sensitivity of IR detectors and experimental noises, the thermal signal of smaller and deeper defects is below the noise level, and STSCS algorithm is unable to recover the defect.

To detect smaller or deeper defects, future work will investigate improvements to PIT hardware for enhancing imaging capabilities, such as strategies to minimize heat losses from the specimen. In addition, we will consider alternative algorithmic approaches for thermography data analysis and image segmentation. In the future work, we will consider a parametric analysis of weak thermal patterns for different signal to noise ratio to establish the limits of PIT detection with STSCS. In this study, the STSCS algorithm performance was optimized using the ground truth knowledge of the calibrated location of defects in the specimens. In the NDE applications, the locations of defects would not be known a-priori. We anticipate mitigating this challenge with a semi-supervised approach^54^, potentially involving development of a foundational model for PIT data analysis^55^. A foundation model can be trained through on-line learning on extensive data to achieve sufficiently high precision and recall scores to allow for using the model in NDE applications.

## Methods

### Overview of pulsed infrared thermography (PIT) imaging

**Figure 4:**
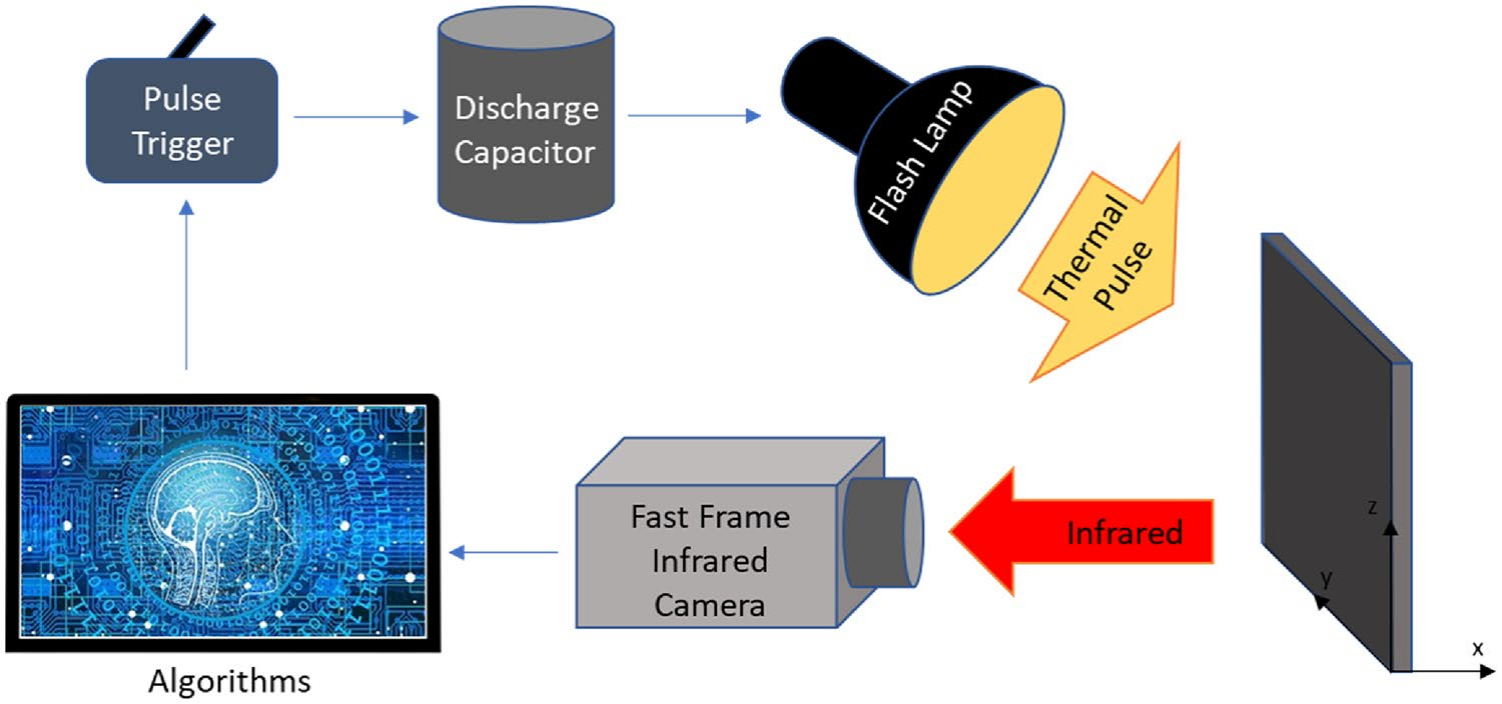
Schematic drawing of pulsed infrared thermography (PIT) data acquisition setup. Flash lamp deposits thermal pulse on material sample surface. Fast-frame IR camera records surface transient temperature as heat is diffusing into the material bulk.

Schematic diagram of Pulsed Infrared Thermography (PIT) system is shown in Fig. 4. The PIT experimental setup includes the IR camera, high energy electrical capacitor, pulse trigger, flash lamp and PC^45^. In PIT, the pulse trigger initializes a high-energy capacitor discharge through a white light flash lamp to rapidly heat the specimen surface. A fast frame IR camera is synchronized to record images of material surface temperature transients. The stack of observed thermography images is acquired and further processed using the UL algorithms to enhance the detection of material defects in images. In the experimental setup, the Balcar ASYM 6400 electrical capacitor is used to deliver a pulse of 6.4 kJ/2 ms thermal energy through the flash lamp to heat the specimen surface. The experimental setup uses FLIR X8501SC MWIR camera with a spectral range of 3–5 µm. The cooled FLIR indium antimonide (InSb) semiconductor allows to achieve thermal sensitivity of 20 mK NETD. The camera can record 180 frames per second at a full 1280 × 1024 pixels resolution. Windowing allows for faster frame rates, up to 29 kHz. The IR camera uses a 50-mm imaging lens and a set of extender rings to obtain different spatial imaging resolutions.

### Development of microscopic flat bottom hole defects in SS316 plates

**Figure 5:**
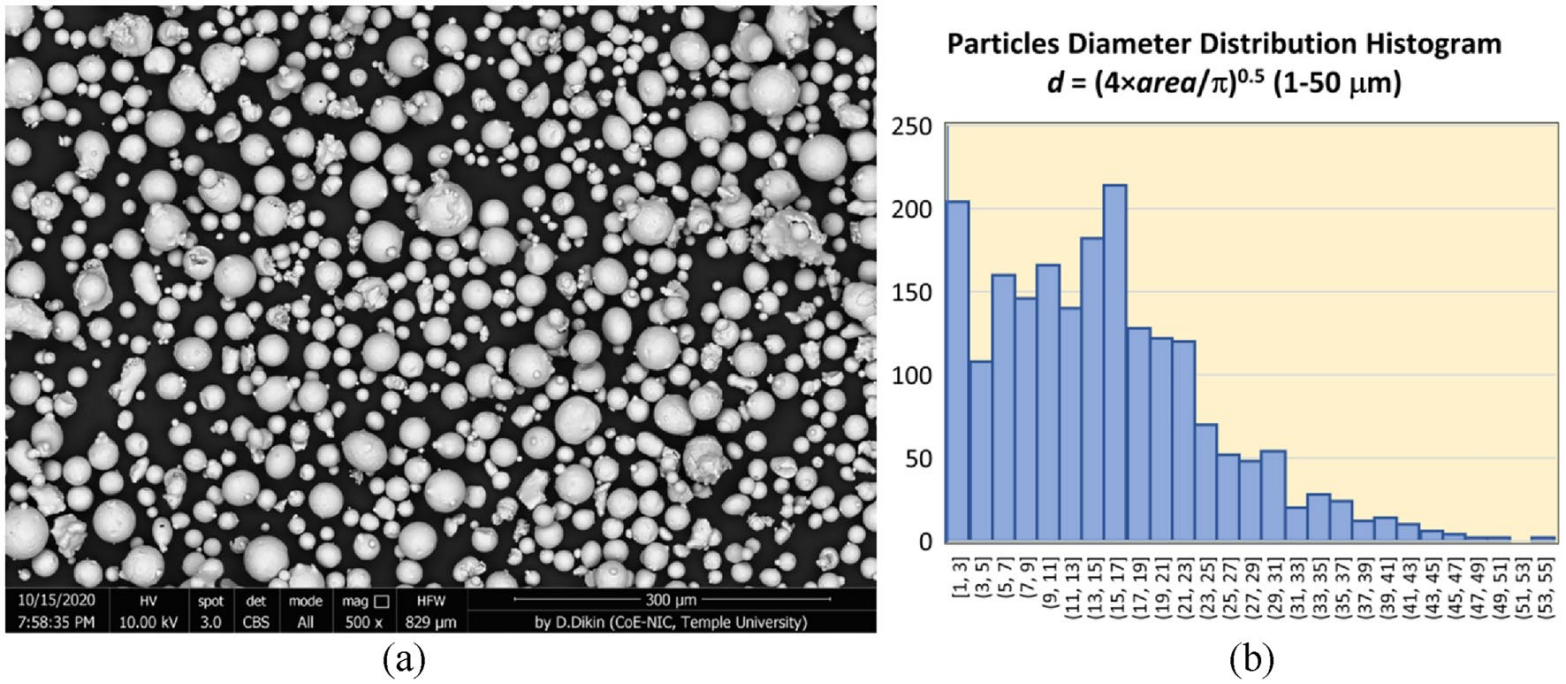
(**a**) SEM micrograph of SS316 TrueForm powder particles. (**b**) Histogram of particle diameter distribution.

In previous studies, we have demonstrated the capability of PIT in imaging calibrated defects with sizes on the order of 1 mm-diameter in SS316^45^. These calibrated defects consisted of imprinted hemispherical regions containing un-sintered powder in AM SS316 specimens, and flat bottom hole (FBH) indentations drilled in SS316 specimens. In this study, we investigate performance of UL in processing PIT images to detect microscopic FBH defects in SS316 specimens. Calibrated FBH defects were introduced into SS316 specimens with electro discharge machining (EDM) drill. Prior studies of PIT performance involved calibrated defects introduced as either FBH defects created in metallic specimens with a high-strength drill, or hemispherical inclusions containing un-sintered trapped powder imprinted into metallic specimens during LPBF process. Creating microscopic calibrated defects in high strength metal is a challenge with LPBF process involves sintering SS316 metallic powder grains with average diameters of 20 µm and 40 µm, respectively. A scanning electron microscope (SEM) micrograph of SS316 TrueForm powder grains is shown in Fig. 5a. The histogram of particle diameter distribution is plotted in Fig. 5b. One can observe that particle diameters can be as large as 50 µm. Creating imprinted porosity defects with diameters smaller than 300 µm with LPBF involves trapping several un-sintered powder grains. Controlling inclusion size at this length-scale is a difficult task because heat diffusion is involved in sintering. In addition, LOF and keyhole inclusion are typically air voids in metal with no trapped powder. Therefore, in developing microscopic calibrated defects, the FBH model of material defect for proof-of-principle studies was selected. FBH defects closely model air voids from a heat transfer physics point of view. FBH’s can be created to be of precise shapes, diameters, and depths relative material surface with an EDM (electron discharge machining) drill.

**Figure 6:**
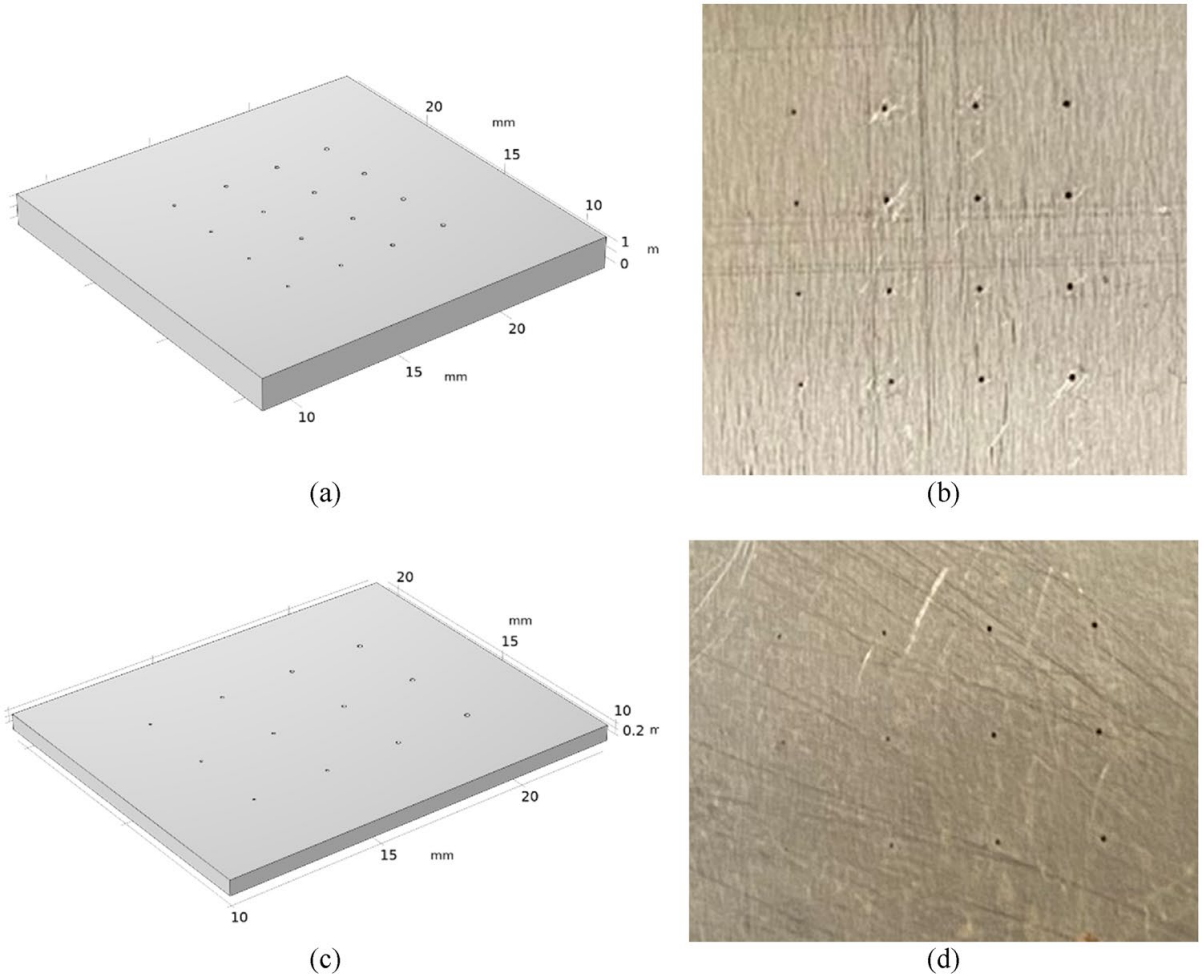
(**a**) Computer visualization of Plate 1 with dimensions 32.9 mm × 32.9 mm × 1.4 mm. (**b**) Photograph of EDM-drilled microscopic FBH’s with sizes from 203 µm to 127 µm in Plate 1. (**c**) Computer visualization of Plate 2 with dimensions 32.9 mm × 30.4 mm × 0.5 mm. (**d**) Photograph of EDM-drilled microscopic FBH’s with sizes from 152 to 76 µm in Plate 2.

In our study, we developed two SS316 plates with microscopic FBH defects produced with EDM drill. In Plate 1 with 32.9 mm × 32.9 mm × 1.4 mm dimensions, microscopic defects with sizes in the range from 203 to 127 µm were introduced at depths in the range from 508 to 127 µm below flat surface. In Plate 2 with 32.9 mm × 30.4 mm × 0.5 mm dimensions, microscopic defects with sizes in the range from 152 to 76 µm were introduced at depths in the range from 381 to 127 µm below flat surface. The two plates have approximately the same surface areas, but the thickness of Plate 2 is almost three times smaller than for Plate 1. Using a thinner Plate 2 allowed for EDM drilling of FBHs with diameters smaller than 127 µm that would come to 127 µm depth below the flat side of the plate. Figure 6a shows the computer visualization of Plate 1. Figure 6b shows the photograph of the back surface of Plate 1 with EDM-drilled microscopic FBH’s with sizes from 203 to 127 µm. Figure 6c shows the computer visualization of Plate 2. Figure 6d shows the photograph of EDM-drilled microscopic FBH’s with sizes from 152 to 76 µm. To the best of our knowledge, these are the smallest FBH defects in stainless steel reported in literature. Thermophysical parameters of SS316L host material are *ρ* = 7954 kg/m^3^, *k* = 13.96 W/mK, *c* = 499.07 J/kg. Thermophysical parameters of air defects are *ρ* = 1.225 kg/m^3^, *k* = 26.24 mW/mK, *c* = 1.00 kJ/kg.

### Spatial–temporal separation-based clustering segmentation (STSCS) algorithm

**Figure 7:**
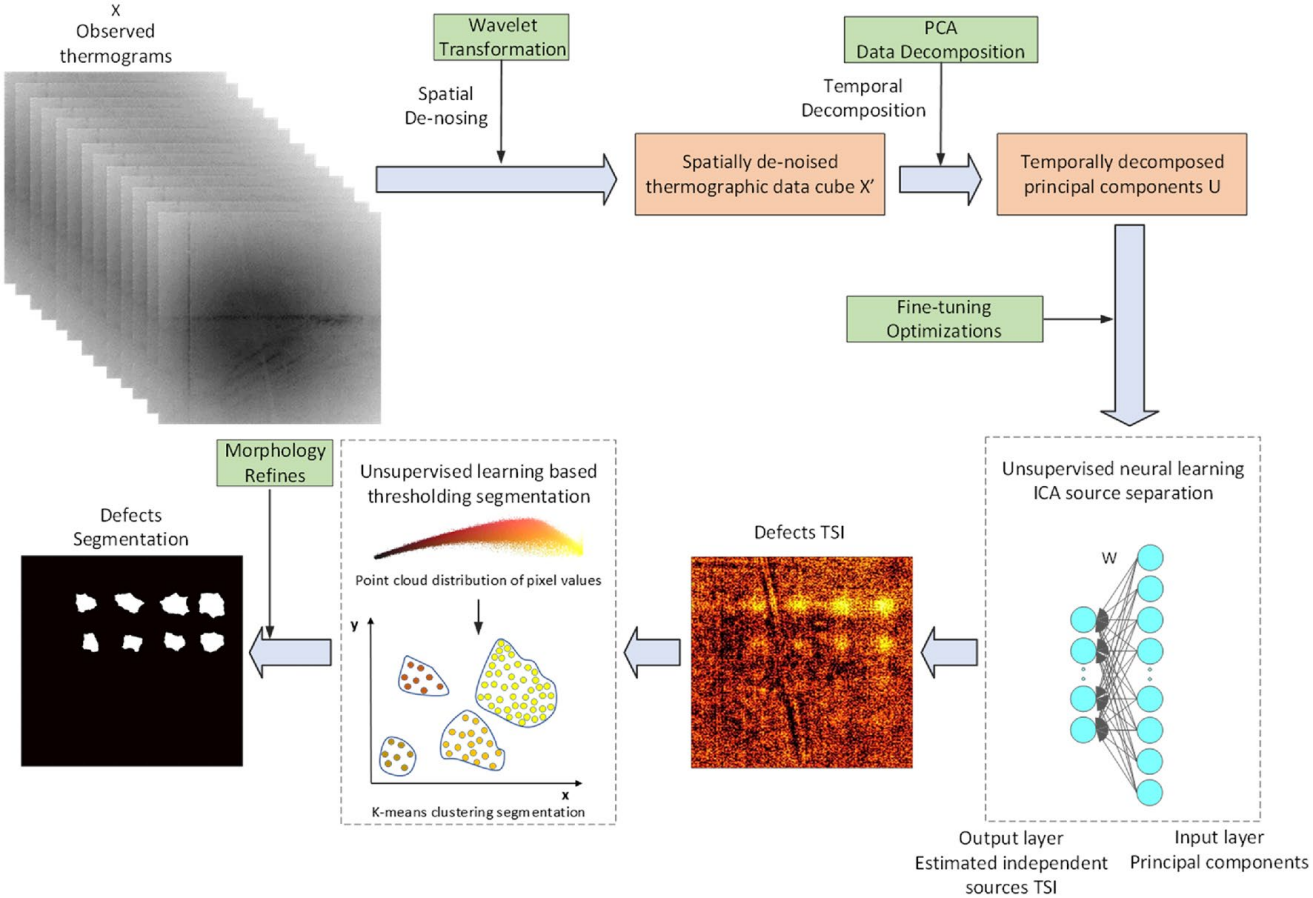
Flow chart of the STSCS algorithm.

The UL spatial–temporal separation-based clustering segmentation (STSCS) algorithm includes four stages of processing of measured thermograms to reduce thermal imaging noises and eliminate data redundancy to recover latent defect patterns. The stages are (1) Wavelet Transformation, (2) Principal Component Analysis (PCA), (3 (3) Independent Component Analysis (ICA), and (4) Segmentation. The flow chart of the STSCS algorithm is shown in Fig. 7.

### Wavelet transformation

The undecimated discrete wavelet transformation^56^ was implemented to spatially de-noise measured thermograms. Wavelet transformation is commonly used for adaptive analysis of time-domain signals with time-dependent frequency. The undecimated feature is advantageous for translation invariance, so that the complete characteristics of signals can be effectively analyzed. This noise is a random variation of brightness or color information in images and normally includes additive white Gaussian noise which can be efficiently reduced by using the wavelet transformation to improve the image resolution. The wavelet analysis determines the appropriate frequency band adaptively and filters the abrupt changes or noise in images to enhance the image quality. The decomposition levels and wavelets of transformation determine the de-noising performance. A higher number of transformation levels represents better de-noising but can lead to erasing of the fine features in images. In this study, we comprehensively benchmarked different transformation levels with various wavelets, and deployed the two-level biorthogonal wavelet transformation, which is advantageous for filtering image noise with minimal impact on image contrast gradients to maintain high image resolution. Wavelet decomposition selects the appropriate frequency band adaptively, then analyzes and filters the high frequency thermography image noise in the spatial domain. Each measured thermogram is decomposed using wavelet transformation into four sub-band images, which include one reconstruction image and three high-pass wavelet images. We preserve the reconstruction images to form a new 3D data cube *X*, and remove the high-pass wavelet images containing most of the image noises.

### Principal component analysis

The PCA processes de-noised thermograms to enhance defect detection by separating defects from the background. The PCA decomposes high-dimensional de-noised thermograms into a few principal thermography components to reduce data redundancy in time. The PCA is trained to find the fewer latent patterns, which are called principal components (PC’s) in high-dimensional thermography data. These PCs include orthogonal dimensions and are obtained by maximizing the variance and minimizing the mean squared error (MSE) between the thermography data cube *X* and reconstructed data. The first PC contains the largest variance of thermography data, and each subsequent PC has an incrementally decreasing contribution to the total data variance. Therefore, the learned PC’s preserve most signatures in thermography data by removing redundant information to enhance the performance in defects detection. We implement the PCA training by deploying the Singular Value Decomposition (SVD) algorithm^57^ which closely resembles PCA. The SVD suffers less from numerical noise because the covariance matrix does not need to be calculated. The equation implementing SVD is:

4 $$ X_{M \times N} = U_{M \times N} R_{N \times N} V_{N \times N}^{T} $$

In Eq. (4), before applying the SVD algorithm, we convert the data cube *X* into a condensed 2D data matrix by transforming the total number of pixels in each thermogram into a singular vector with the time-dependent information into columns. *M, N* represent the number of rows, and columns of the condensed 2D data matrix *X*, and *N* is the number of observed thermograms. Therefore, in PCA training, the SVD decomposes de-noised thermograms *X* into principal thermography components *U*. Here *U* and *V* denote the orthogonal matrices showing the variability of data and time information in thermography data, respectively. In Eq. (1), *R* is the matrix containing the singular values corresponding to *U*. The de-noised thermograms can be reconstructed with few principal thermography components of *U* to train the ICA as inputs to enhance the performance for defects detection.

### Independent component analysis

In the PIT measurements, the thermograms consist of different independent thermography signals that correspond to surface regions of defects, non-defects, and noise. All of these signals demonstrate different temporal evolutions. During the transients, the evolution of the surface temperature for each region of the specimen displays the non-Gaussian distribution. However, the distribution of the sum of all thermography signals (independent source signals) approaches Gaussian distribution regardless of the distribution for each thermography signal^51^. Therefore, in the 3rd stage, we train the neural learning-based ICA using learned principal thermography components. This is accomplished by estimating the separation matrix *W* to maximize the non-Gaussian distribution. This separation matrix discretizes each independent thermography signal from the mixed thermograms to reconstruct the Thermal Source Image (TSI)^44^ for defects detection. We applied Eq. (2) as the objective function *O(w)* to approximate the Negentropy^51^ to measure the non-Gaussian content:

5 $$ O\left( w \right) = \left[ {E\left\{ {G\left( {w^{T} U^{T} } \right)} \right\} {-} E\left\{ {G\left( g \right)} \right\}} \right]^{2} $$

6 $$ E\left\{ {\hat{S}\hat{S}^{T} } \right\} = WE\left\{ {\hat{U}\hat{U}^{T} } \right\}W^{T} = WW^{T} = I $$

In Eq. (5), *U* represents the principal thermography components and *w* is a unit vector in the separation matrix *W*. *G* is any nonquadratic function for performance optimization. We aim to find the projection *w*^*T*^*U*^*T*^ to maximize the non-Gaussian content to estimate each independent source signal. In this study, each unit vector *w* is optimized as a neuron updated with the learning rule. For separation of all source signals, the matrix *W* is estimated as multiple output neurons in training. We deployed the fast-fixed point algorithm^51^ for optimization to obtain *W* for reconstruction of defects TSI. To enhance the performance in defect detection, the estimated source signals $\hat{S}$, which are TSI of defects, are constrained to be uncorrelated under Eq. (6) ^51^. In training, each unit vector *w* is iteratively updated to maximize the objective function *O(w).* If the convergence is satisfied, which indicates that the updated *w is* pointing in the same direction as the previous *w*, we use this unit vector *w* to estimate the TSI to detect defects. Otherwise, we use the updated *w* to initiate another iteration of training.

To enhance the ICA training performance, we implemented the fine-tuning process with additional optimizations using adaptive learning rate method and the hyperbolic tangent as nonquadratic function. Adaptive learning rate method improves model performance by automatically altering learning rates during training. This allows the model to adapt to changes in optimization for better generalization and faster convergence. In this approach, the learning rate is rapidly halved to stabilize the learning process. The hyperbolic tangent function results in higher values of gradient during training to enable the learning process to be more efficient. In addition, the model was trained to maximize defects detectability while obtaining the highest compression ratio using the minimal number of latent dictionaries.

### Segmentation

Image segmentation aims to separate the pixels of interest from the background by creating boundaries and locating objects^58^. In stage 4, we further optimize visualization of defects in TSI by developing the UL-based graph thresholding for defects segmentation. This thresholding technique utilizes the K-means clustering algorithm^59^ to efficiently segment defects from the imaging background despite the presence of low contrast gradients. The K-means clustering algorithm is a UL method to separate groups of objects by searching for partitions, so that the objects within the same cluster are as close to each other as possible. The threshold values are determined in the following steps: 1. Indicate the number of clusters; 2. Assign pixel color values randomly to any of the clusters; 3. Calculate cluster centroid; 4. Calculate distance of pixel color values from cluster centroid; 5. Reassign pixel color values to the nearest clusters based on distance of pixel color values from cluster centroid; 6. Calculate new cluster centroid; 7. Repeat steps: 4, 5, 6 until there is no update of pixel color values in clusters. In training, the graph thresholding process deploys the K-means clustering algorithm to iteratively group pixels by similar attributes, such as pixel color values, into the same clusters for thresholding to segment defects from the background. If the convergence is satisfied, so that the same pixels are consistently assigned to one cluster while thresholding is updated, we use this thresholding value to segment defects by creating a pixel-wise mask. The segmentation results reduce blurring of edges and demonstrate minimal errors to changes in threshold values. Therefore, we refine the defects segmentation by morphology image processing methods to enhance the visibility of defects. The morphology method adjusts each pixel based on the neighboring pixels, which is implemented as a dilation followed by erosion operations^60^. The dilation operation compensates for false negative outcomes from the graph thresholding process by filling small holes in the segmentation image, while the erosion operation compensates for false positive outcomes by removing floating pixels.

### Online learning

**Figure 8:**
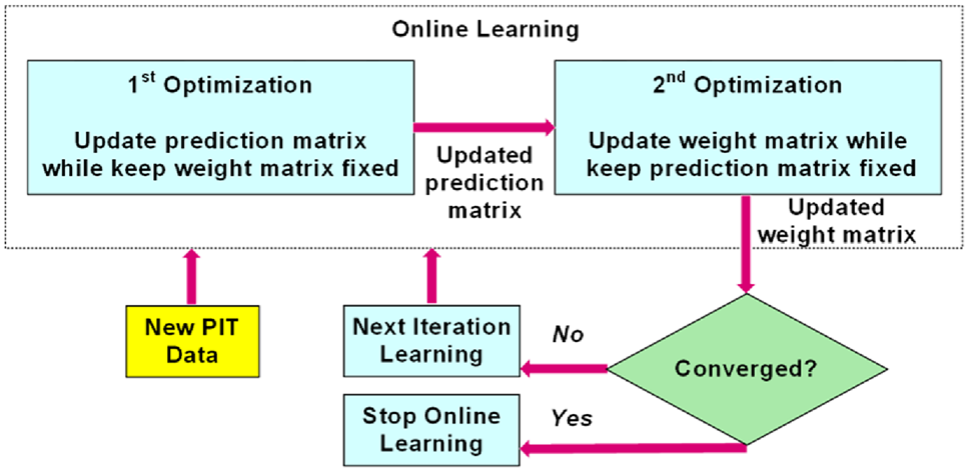
Flow chart of unsupervised online learning in STSCS.

The STSCS algorithm includes the online learning feature based on unsupervised incremental learning on PIT data mini-batches. The online method allows the STSCS model to periodically acquire new information whenever a new training set becomes available, while preserving the knowledge learned in previous training data sets. The flow chart of unsupervised online learning in STSCS is shown in Fig. 8. The online learning process involves a dual optimization mechanism^61^ to train the STSCS model. In training, the prediction matrix and weight matrix are iteratively updated to optimize the objective function. If the convergence is not satisfied for the new PIT data, another iteration of training is initiated with updated matrices. Otherwise, a new re-trained STSCS model is generated to process new PIT data.

## Data Availability

The datasets used and/or analyzed during the current study available from the corresponding author on reasonable request.
